# Supplementary material for: Identification of photoperiod-induced specific miRNAs in the adrenal glands of Sunite sheep (Ovis aries)
Source: Front Vet Sci. 2022 Jul 22;9:888207. doi: 10.3389/fvets.2022.888207 (PMC9354845; doi:10.3389/fvets.2022.888207)
Supplement: Supplementary file 1 [file Data_Sheet_1.ZIP › Supplementary file/miRDeep2 file.zip!/miRDeep2 file/Novel_Structure/SPLP42A2/Novel_530.pdf]

Provisional ID	:	NC_019466.2_19415
Score total	:	51.2
Score for star read(s)	:	3.9
Score for read counts	:	44
Score for mfe	:	1.7
Score for randfold	:	1.6
Score for cons. seed	:	
Total read count	:	98
Mature read count	:	92
Loop read count	:	0
Star read count	:	6

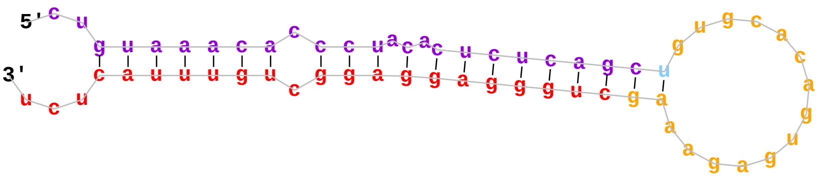

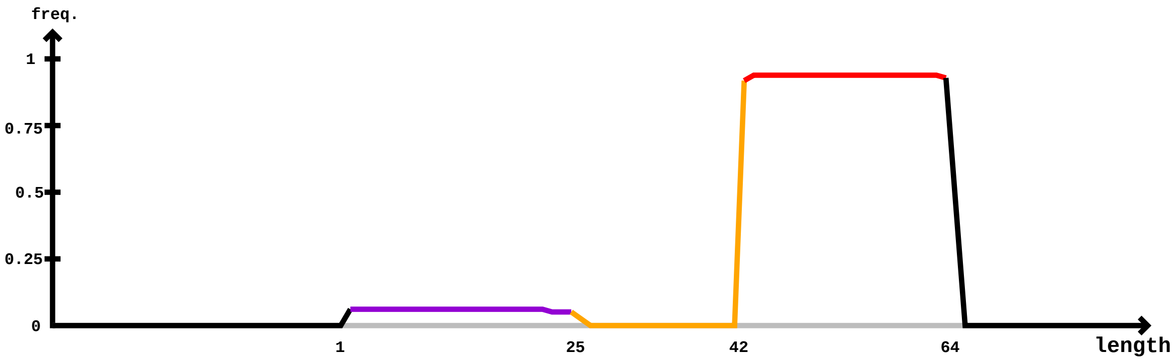

Star Mature

```
5'- ccgccccagagagcacugagggccagauacuguaaacacccuacacucucagcugugcacagugagaaagcugggaggaggcuguuuacucucucugccuuggaagucaacu   -3'    obs
    ccgccccagagagcacugagggccagauacuguaaacacccuacacucucagcugugcacagugagaaagcugggaggaggcuguuuacucucucugccuuggaagucaacu          exp
    ..(((((((......))).))))((((....(((((((.(((.(.(((((((((...............))))))))))))).)))))))....))))..............    reads      mm         sample
    .............................cuguaaacacccuacacucuc..............................................................    1          0          seq
    .............................cuguaaacacccuacacucucagc...........................................................    4          0          seq
    .............................cuguaaacacccuacacucucagcu..........................................................    1          0          seq
    ......................................................................cugggaggaggcuguuuacuc.....................    1          0          seq
    ......................................................................cugggaggaggcuguuuacucu....................    87         0          seq
    ......................................................................cugggaggaggcuguuuacucuc...................    2          0          seq
    .......................................................................ugggaggaggcuguuuacucu....................    2          0          seq
```
